# Characterization of fluoroquinolone resistance and *qnr* diversity in *Enterobacteriaceae* from municipal biosolids

**DOI:** 10.3389/fmicb.2013.00144

**Published:** 2013-06-11

**Authors:** Ella Kaplan, Maya Ofek, Edouard Jurkevitch, Eddie Cytryn

**Affiliations:** ^1^Department of Soil Chemistry, Plant Nutrition and Microbiology, Institute of Soil, Water and Environmental Sciences, The Volcani Center, Agricultural Research OrganizationBeit Dagan, Israel; ^2^Department of Agroecology and Plant Health, The Robert H. Smith Faculty of Agriculture, Food and Environment, The Hebrew University of JerusalemJerusalem, Israel

**Keywords:** activated sludge, biosolids, fluoroquinolone, ciprofloxacin, antibiotic resistance, *qnr*, integron

## Abstract

Municipal biosolids produced during activated sludge treatment applied in wastewater treatment plants, are significant reservoirs of antibiotic resistance, since they assemble both natural and fecal microbiota, as well as residual concentrations of antibiotic compounds. This raises major concerns regarding the environmental and epidemiological consequences of using them as fertilizers for crops. The second generation fluoroquinolone ciprofloxacin is probably the most abundant antibiotic compound detected in municipal biosolids due to its widespread use and sorption properties. Although fluoroquinolone resistance was originally thought to result from mutations in bacterial gyrase and topoisomerase IV genes, it is becoming apparent that it is also attributed to plasmid-associated resistance factors, which may propagate environmental antibiotic resistance. The objective of this study was to assess the impact of the activated sludge process on fluoroquinolone resistance. The scope of resistances and mobile genetic mechanisms associated with fluoroquinolone resistance were evaluated by screening large collections of ciprofloxacin-resistant *Enterobacteriaceae* strains from sludge (*n* = 112) and from raw sewage (*n* = 89). Plasmid-mediated quinolone resistance determinants (*qnrA*, *B*, and *S*) were readily detected in isolates from both environments, the most dominant being *qnr*S. Interestingly, all *qnr* variants were significantly more abundant in sludge isolates than in the isolates from raw sewage. Almost all ciprofloxacin-resistant isolates were resistant to multiple antibiotic compounds. The sludge isolates were on the whole resistant to a broader range of antibiotic compounds than the raw sewage isolates; however, this difference was not statistically significant. Collectively, this study indicates that the activated sludge harbors multi-resistant bacterial strains, and that mobile quinolone-resistance elements may have a selective advantage in the activated sludge.

## INTRODUCTION

Municipal biosolids produced during the activated sludge process in secondary wastewater treatment are often utilized as fertilizers for a wide array of crops, due to their availability and high nutrient content (Dröge et al., 2000; Golet et al., 2003). However, despite the obvious advantages of this practice, sludge application may have highly negative ecological and epidemiological ramifications. Wastewater treatment plants (WWTPs) represent an endless pool of commensal human and farm animal bacteria (Dröge et al., 2000; Zhang et al., 2009), which are constantly exposed to a wide range of anthropogenic compounds, such as antibiotics. These compounds can select for resistant strains of both pathogenic and non-pathogenic bacteria, by means of both vertical and horizontal gene transfer (Pellegrini et al., 2011).

The application of municipal biosolids to soil may not only result in transfer of resistant bacterial strains, but also in dissemination of antibiotic resistance genes (ARGs) that can enhance environmental antibiotic resistance reservoirs. Indeed, there is growing perception that ARGs are emerging contaminants with the ability to spread from anthropogenic sources, such as WWTPs, into natural environments (LaPara et al., 2011; Li et al., 2012; Pruden et al., 2012). Although municipal biosolids are generally stabilized before application to soils, a recent study detected significant levels of ARGs in biosolids subsequent to different stabilization methods (Ma et al., 2011; Munir and Xagoraraki, 2011), and elevated levels of these genes were even observed in soil samples after land application of biosolids (Munir and Xagoraraki, 2011).

Fluoroquinolones are fully synthetic broad-spectrum antibacterial agents that are becoming increasingly popular in the treatment of clinical infections. The mechanism by which fluoroquinolones inhibit cell proliferation was well elaborated by Jacoby (2005) and by Strahilevitz et al. (2009).

Ciprofloxacin is a second generation fluoroquinolone and is the fifth most commonly prescribed antibacterial with over 20 million outpatient prescriptions written in the US alone in 2010. The metabolism of ciprofloxacin in the human body is only partial and so it is excreted in both urine (45–62%) and feces (15–25%), and transported to WWTPs through municipal sewage systems (Golet et al., 2003). During wastewater treatment, a large fraction of ciprofloxacin is removed from the aqueous phase and absorbed into the sludge, thereby accumulating in dewatered sludge, where concentrations of up to 50 mg/kg dry weight have been detected (Golet et al., 2002; McClellan and Halden, 2010).Field experiments have demonstrated that sorption of fluoroquinolones to municipal sludge results in long-term persistence, which continues even after application to agricultural soils (Golet et al., 2003).

When fluoroquinolones were first introduced for clinical use in the mid 1980s the likelihood for the emergence of resistance was considered to be negligible, because bacteria would have to spontaneously acquire two or more non-fatal mutations in the catalytic sites of the gyrase/topoisomerase IV enzymes to evade the antibiotic effect (Robicsek et al., 2006). Nonetheless, shortly after fluoroquinolones became one of the top antibiotic compounds in nosocomial use, resistance became a common global phenomenon (Robicsek et al., 2006; Strahilevitz et al., 2007). Originally, fluoroquinolone resistance was attributed to mutations in specific areas of DNA gyrase genes (*gyrA*, *parC*, and *parE*), known as quinolone resistance-determining regions. However, in the late 1990s emerging evidence indicated acquisition of fluoroquinolone resistance in a non-clonal manner (Robicsek et al., 2006), with the detection of a plasmid-mediated-quinolone-resistance mechanism by Martinez-Martinez et al. (1998). This plasmid encoded a pentapeptide-repeat protein termed QnrA (for quinolone resistance) that increased quinolone resistance levels between 8- and 64-fold in *Escherichia coli*. Although the resistance of these isolates was approximately 10-fold lower than traditional mutation-associated minimal inhibitory concentration (MIC), they were found to have increased probabilities for acquisition of additional resistance mechanisms (Tran and Jacoby, 2002). Experiments conducted with a purified, His6 QnrA determined that it eliminated the quinolone action by directly binding to gyrase and preventing the antibiotic from binding to the DNA, thus being trapped in the lethal gyrase–DNA–quinolone cleavage complex (Tran and Jacoby, 2002; Tran et al., 2005; Strahilevitz et al., 2009).

The worldwide expansion of *qnr* was rapid. In a study conducted in Alabama in 1994 by Martinez-Martinez et al. (1998), the newly defined *qnr*A variant was identified in only one host-associated *Klebsiella pneumoniae* isolate, and was not detected among any other of 350 Gram-negative isolates that were screened over 6 months. Ten years later, the prevalence of *qnr*A among ciprofloxacin-resistant *K. pneumoniae* isolates rose to 11% (in a 3-year survey done in six US states; Wang et al., 2004). During this period of time, *qnr*A-like determinants were also found in 7.7% of ciprofloxacin-resistant *E. coli* isolates in Shanghai, China (Wang et al., 2003). In light of these, and many other studies, it may be suggested that the increase in fluoroquinolone resistance was driven by the increase in *qnr* prevalence, which is horizontally transferred and confers a selective advantage to plasmid bearing bacteria (Martinez-Martinez et al., 1998; Nordmann and Poirel, 2005; Strahilevitz et al., 2007).

Several other variants of the Qnr protein have been discovered to date, including QnrB, QnrS, and the rarer QnrC and QnrD. All Qnr proteins involve a similar mechanism of protecting the bacterial gyrase/topoisomerase IV from the quinolone, and vary in size from 214 to 221 amino acids (Strahilevitz et al., 2009). Other studies show that although originating in different bacterial hosts, *qnr* genes are mostly found adjacent to sulfonamide resistance genes – *sul-I* and *sul-II*, and are often carried on class-1 integrons, in proximity to the integrase (*intl-1*) gene, which are prevalent in a wide range of ecological habitats (Tran and Jacoby, 2002; Strahilevitz et al., 2009; Pellegrini et al., 2011).

The objective of this study was to assess the phenotypic and genotypic scope of quinolone resistance in raw sewage and in WWTP sludge and to determine whether quinolone resistant bacteria, and specifically if plasmid-associated quinolone-resistance genes are enriched in WWTPs during sewage treatment. We isolated a large collection of ciprofloxacin-resistant *Enterobacteriaceae* from both the raw sewage entering the WWTP, and from dewatered sludge, and these isolates were screened for *qnrA*, *B*, and *S* variants, and for class-1 integron-associated integrase (*intl-1*) genes. Ciprofloxacin-resistant isolates were also screened against 1 μg/ml ciprofloxacin and six other antibiotic compounds to assess levels of multi-resistance in these environments. The initial isolation of the analyzed *Enterobacteriaceae* was conducted on 0.4 μg/ml ciprofloxacin, which corresponds to intermediate ciprofloxacin (IC) resistance that is a characteristic of *qnr*-harboring isolates. This is approximately 2.5-fold below MIC values associated with gyrase/topoisomerase IV mutant strains (Hopkins et al., 2005; Nordmann and Poirel, 2005; Gosling et al., 2012).

## MATERIALS AND METHODS

### SITE DESCRIPTION AND SAMPLING PROTOCOL

The Dan Region Wastewater Treatment and Reclamation Project (termed Shafdan), is the largest and most complex wastewater treatment facility in Israel. It treats approximately 340,000 m^3^ of sewage daily from the entire metropolitan Tel Aviv area including five major hospitals. Secondary treatment is conducted in 12 activated sludge reservoirs with retention times ranging from 12–14 h. Approximately 15,000 m^3^ of sludge are produced daily (1% dry weight).

Triplicate dewatered sludge and raw sewage samples were taken from the Shafdan WWTP in three individual sampling profiles (April 2012, May 2012, and October 2012). Dewatered sludge (18% water content) was sampled in sterile 100 ml test tubes, and the raw sewage was collected from WWTP influent in 1 l bottles. Samples were transferred to the lab on ice within 1 h, and raw sewage samples were concentrated by centrifuging for 10 min at 1000 *g*.

### *ENTEROBACTERIACEAE* ISOLATION AND CHARACTERIZATION

Five grams of dewatered activated sludge or raw sewage samples were suspended in 40 ml of sterile 0.1 M ammonium phosphate buffer [(NH_4_)_2_HPO_4_], shaken laterally at 350 rpm for 45 min, at room temperature, and centrifuged twice for 10 min at 500 rpm; each time supernatant was transferred into new 50 ml sterile tubes and pellet was discarded. Supernatant was then centrifuged for 10 min at 8,000 rpm, and the pellet was resuspended in 20 ml sterile saline solution. Samples were serially diluted and filtered onto 25 mm, 0.45 μm pore size polycarbonate membranes (GE Healthcare, Chalfont St. Giles, UK). Filters were aseptically placed on 45 mm Petri dishes containing modified membrane thermotolerant *Escherichia coli* (mTEC) agar, which selects for *Enterobacteriaceae*. The modified mTEC method www.epa.gov/nerlcwww/documents/1603sp02.pdf uses the chromogen 5-bromo-6-chloro-3-indolyl-β-D-glucuronide (Magenta Gluc) as the differential agent (Francy and Darner, 2000).

Serial dilutions were platted on media with or without IC concentrations (0.4 μg/ml). This sub-MIC value was selected, because, as mentioned above, it is believed to be a ciprofloxacin concentration which will allow us to screen for *Enterobacteriaceae* that harbor plasmid-mediated fluoroquinolone resistance (Hopkins et al., 2005; Gosling et al., 2012). Plates were incubated for 2 h at 37°C and then for 20–22 h at 45°C as previously described (Francy and Darner, 2000).

The taxonomic affiliation of the isolates was determined by plating on CHROMagar Orientation medium agar plates (CHROMagar Microbiology, Paris, France). CHROMagar typing indicated that all of the *Enterobacteriaceae* isolates from both the raw sewage and the sludge biosolids were affiliated with either the *E. coli* (16.1% of sludge isolates and 11.2% of raw sewage isolates) or *Klebsiella* (78.6% of sludge isolates and 70.8% of raw sewage isolates) genera.

There was no statistical difference in the ration of *E. coli* to *Klebsiella* genera in the sludge relative to the raw sewage.

All of the non-*Enterobacteriaceae* isolates were not used for further analysis. The taxonomic affiliation of 20 of the isolates was further assessed by phylogenetic analysis of partial 16S rRNA gene fragments. Standard Sanger sequencing was conducted at HyLabs (Rehovot, Israel) and the taxonomic affiliation of the sequences was determined by basic local alignment search tool (BLAST). In total, 201 sub-MIC *Enterobacteriaceae* isolates were obtained (112 from the activated sludge, and 89 from the raw sewage), and further analyzed as described below.

### RESISTANCE PHENOTYPING

Ninety activated sludge and 87 raw sewage, sub-MIC ciprofloxacin-resistant isolates were screened for resistance to other antibiotic compounds as follows: isolates were transferred aseptically to Müller-Hinton (MH)-agar containing one of the eight different antibiotics at European Committee on Antibiotic Susceptibility Testing (EUCAST) clinical MIC breakpoint concentrations according to standard procedures (http://www.eucast.org/clinical_breakpoints): ciprofloxacin (1 μg/ml), ampicillin (8 μg/ml), ceftriaxone (2 μg/ml), chloramphenicol (8 μg/ml), and gentamicin (4 μg/ml). In addition, isolates were also screened against nalidixic acid (32 μg/ml) and tetracycline (30 μg/ml). Plates were incubated at 37°C overnight, and colony formation was assessed.

### DNA EXTRACTION FROM ISOLATES

DNA was extracted from all samples using a modified bead-beating method. Bacteria cells were grown overnight in 5 ml Luria-Bertani (LB) broth containing ciprofloxacin (0.4 μg/μl). After harvest, cells were lysed in extraction buffer [100 mM Tris–HCl, pH 8.0; 100 mM potassium phosphate buffer pH 8.0; 1% cetyltrimethylammonium bromide (CTAB); and 2% sodium dodecyl sulfate (SDS)] followed by bead-beating (in the Fast Prep FP 120, Bio101, Savant Instruments Inc., Holbrook, NY, USA). The crude extracts were mixed with KCl to a final concentration of 0.5 M, incubated for 5 min, and centrifuged. DNA present in the supernatant was bound to glassmilk (0.5–10 μm silica particles, Sigma Chemical Co., St. Louis, MO, USA) with 6 M NaI. The silica was then resuspended in an ethanol-based wash buffer solution (Boyle and Lew, 1995) and transferred to a centrifuge tube filter (0.22 μm cut-off nylon filter, Costar, Corning Inc., Corning, NY, USA) that bound the DNA to the silica and retained it on the filter until eluted with 60°C Tris–EDTA (TE) into a sterile tube. Extracted DNA samples were stored at -20°C until use.

### PCR DETECTION OF *qnr* VARIANTS AND *intl-1* GENES

Detection of the *qnr* variants was accomplished by means of a modified multiplex, touchdown PCR protocol based on a previously described method and primers (Cattoir et al., 2007). Briefly, the protocol involved 20 initial cycles: 5 min 94°C, 30 s 94 °C, 30 s 60–50 °C (each cycles reducing 0.5 °C), 20 s 72 °C, repeating the latter three steps; 15 additional cycles: 30 s 94 °C, 30 s 50 °C, 20 s 72 °C; and a final cool down step for 10 min, at 72 °C followed by a slow descent to 10 °C.

For detection of the *intl-1* gene, the following PCR protocol was conducted, using previously reported primers (Gaze et al., 2011): 25 cycles: 3 min 95 °C, 1 min 95 °C, 30 s 58 °C, 10 s 72 °C repeating the latter three steps, 10 min 72 °C and a slow descent to 10 °C. Both PCR programs were executed in a final volume of 12.5 μl containing: 8 ng of purified DNA, 5 U Dream-Taq polymerase (MBI Fermentas, Lithuania), 1× Dream-Taq Buffer, 1.5 mM MgCl_2_, 200 μM dNTPs, 0.4 μM primers.

### STATISTICAL ANALYSES

Statistical analyses were conducted using the Statistica software application (StatSoft, Inc., Tulsa, OK, USA). Multiple statistical comparisons were conducted using the Tukey’s HSD (honestly significant difference) test, in which mean values that do not share any letters are considered significantly different. Pairwise comparisons were conducted using the Fisher’s exact test. Compared values were considered statistically significant when *p* values were less than 0.05.

## RESULTS

### ESTIMATION OF CIPROFLOXACIN-RESISTANT *ENTEROBACTERIACEAE*

**FIGURE 1:**
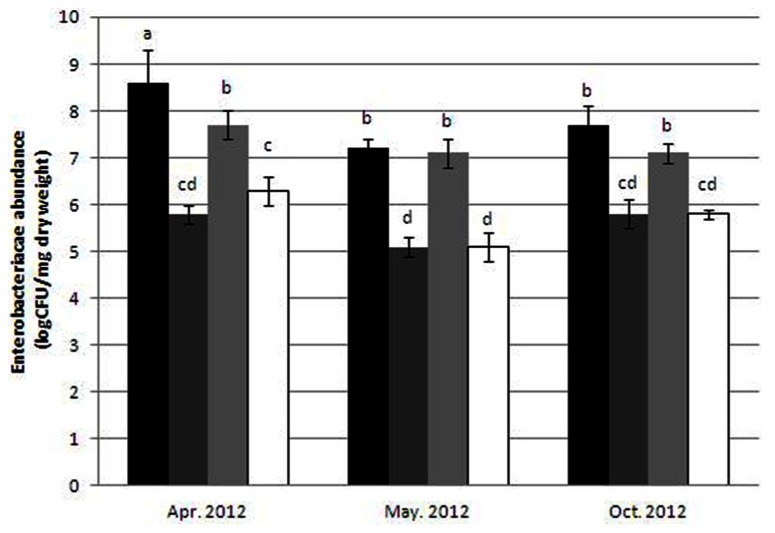
**Abundance of *Enterobacteriaceae* in dewatered sludge: non-selective media (black bars) and 0.4 μg/ml ciprofloxacin-amended media (dark gray bars); and in raw sewage: non-selective media (light gray bars), and 0.4 μ g/ml ciprofloxacin-amended media (white bars)**. Error bars indicate standard deviation. Mean values that do not share any letters are considered significantly different (Tukey’s HSD *p* < 0.05).

*Enterobacteriaceae* levels in both raw sewage and dewatered, activated sludge ranged between 10^7^ and 5 × 10^8^ CFU/mg. Resistance to IC concentrations (0.4 μg/ml) reduced the number of colony forming units in both sample types by approximately two orders of magnitude (**Figure 1**), with no significant differences in the relative abundances of IC-resistant isolates in the sludge in comparison to the sewage isolates.

### SCREENING FOR MULTI-RESISTANCE

**FIGURE 2:**
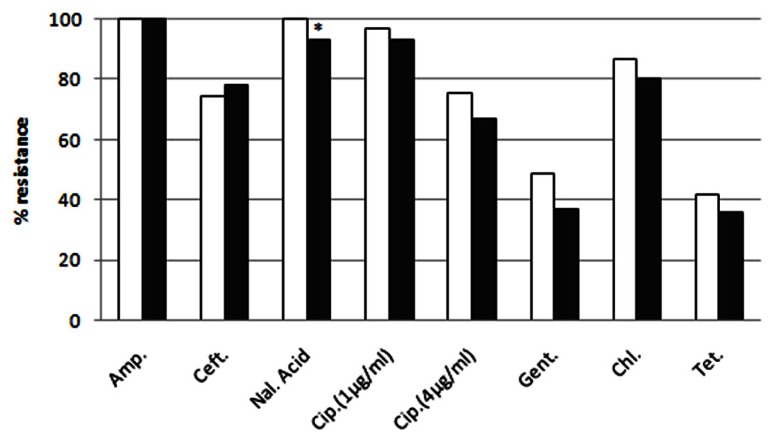
**Resistance of sludge (white bars) and raw sewage (gray bars) intermediate ciprofloxacin-resistant isolates to additional antibiotic compounds at MIC concentrations**. Asterisk indicates statistically significant (Fisher’s exact test *p* < 0.05) differences between sampling locations.

**FIGURE 3:**
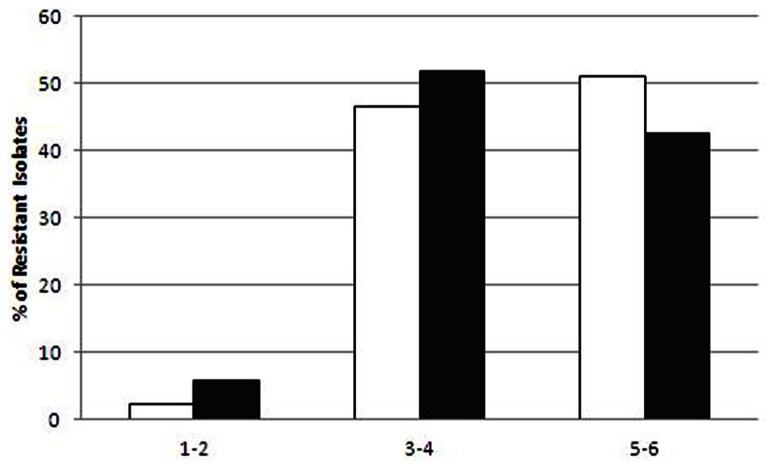
**Level of multi-resistance in intermediate ciprofloxacin-resistant isolates from sludge (white bars) and raw sewage (gray bars)**. The *x*-axis shows the abundance of the strains resistant to 1–2, 3–4, and 5–6 additional antibiotic compounds.

The raw sewage and activated sludge *Enterobacteriaceae* isolates were highly resistant to EUCAST clinical MIC breakpoint concentrations (**Figure 2**). With the exception of ceftriaxone, the ratio of IC isolates resistant to additional antibiotic compounds (at MIC concentrations) was generally higher in the dewatered sludge than in the raw sewage; however, this was only statistically significant for nalidixic acid. The efficacy of different antibiotics against the sewage and sludge isolates was highly similar for all of the antibiotics tested, as shown in **Figure 2**. Additional discrepancies were observed in the resistance profiles of the two environments when examining the scope of multi-resistance of the IC isolates. The sludge IC isolates were generally resistant to a larger range of antibiotic compounds; however, this was not statistically significant (**Figure 3**).

### DETECTION OF *qnr* VARIANTS AND PRESENCE OF CLASS-1 INTEGRONS

**FIGURE 4:**
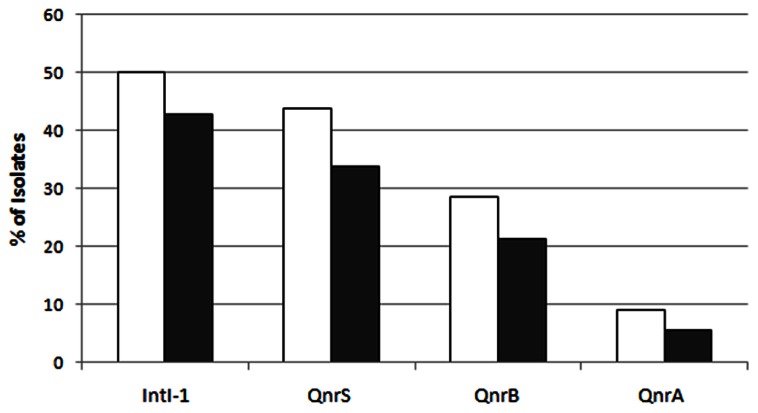
**The abundance of the different variants of *qnr* and of the *intl-1* genes in sludge (white bars) and raw sewage (gray bars) intermediate ciprofloxacin-resistant isolates**.

In total, 75% of the sludge IC isolates and 59.6% of the raw sewage IC isolates were found to harbor at least one *qnr* variant. This suggests a possible enrichment of plasmid-mediated quinolone resistance in the activated sludge (*p* < 0.05). The *qnrS* variant was the most abundant, detected in 43.8 and 33.7% of the sludge and raw sewage IC isolates, respectively; the *qnrB* variant accounted for 28.5 and 21.3%, respectively; and *qnrA* was detected in 9 and 5.6%, respectively (**Figure 4**).

**FIGURE 5:**
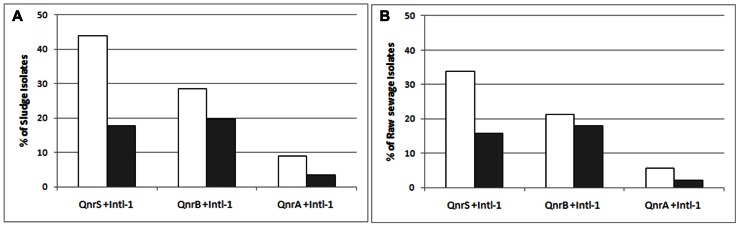
***White bars*: the relative abundance of intermediate ciprofloxacin-resistant isolates carrying *qnr* variants (A, B, S) from the sludge (A), and raw sewage (B); *Gray bars*: the abundance of *intl-1* positive isolates among those *qnr*-positive isolates**.

The *intl-1* gene was found in 50 and 42.7% of sludge and raw sewage IC isolates, respectively (**Figure 4**). Although *qnr*S was the most prevalent variant detected in both sludge and raw sewage, *qnr*B was found to have the strongest association with *intl-1* (**Figure 5**).

Contingency tests were conducted on the question whether specific populations of bacteria (*E. coli* and *Klebsiella*) are more or less prone to carry the different *qnr* variants or the *intl-1* gene. No such correlation was found.

**FIGURE 6:**
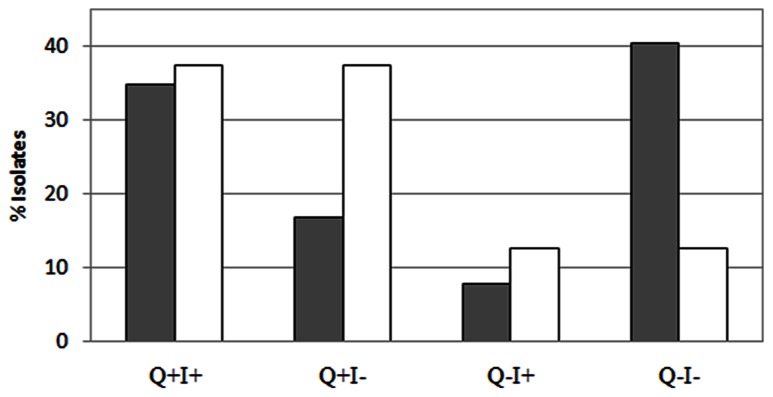
**Correlation between *qnr* and *intl-1* presence: isolates exhibited a negative correlation between *qnr* (Q) and their *intl-1* (I) content in sludge (white bars) and raw sewage (gray bars)**.

Several studies have found that *qnr* genes are associated with class-1 integrons. Therefore, the linkage between *qnr* and *intl-1* genes was more comprehensively explored. Approximately 81.5% of the raw sewage *intl-1*-positive IC isolates were *qnr* positive (I^+^Q^+^), whereas only 29.4% of the *intl-1*-negative IC isolates harbored *qnr* (I^-^Q^+^). This stands in complete contrast to the activated sludge, where 75% of both the I^+^ and I^-^ IC isolates contained *qnr* genes (**Figure 6**).

## DISCUSSION

Wastewater treatment plants are man-made ecological niches that assemble high concentrations of fecal and environmental bacteria. These giant “chemostats” have the potential to serve as hotspots for horizontal gene transfer. Horizontal transfer of ARGs in activated sludge is an actual concern, due to the potential evolution of multi-resistance strains, which are later discharged into agricultural environments when municipal biosolids are used as fertilizer. These ARGs can readily enter water supplies and the food chain, and henceforth may have significant epidemiological ramifications. Bacterial pathogens from municipal biosolids are generally not considered as high risk factors for disease propagation, since the sludge is generally stabilized by anaerobic digestion or compostation prior to application as fertilizer. However, a fraction of sludge bacteria, and more notably, horizontally transferable genetic entities harboring ARGs can survive sludge stabilization processes and this gene pool may be disseminated into the soil microbiome following municipal sludge application.

In this study we focused on plasmid-mediated quinolone resistance (Qnr) determinants. Although these determinants have been found to occur on chromosomal DNA in certain environmental bacteria (Poirel et al., 2005), they are considered to be almost exclusively plasmid-borne in *Enterobacteriaceae* (Rodriguez-Martinez et al., 2011). This characteristic is especially concerning due to the clinical importance of fluoroquinolones and the rapid spread of plasmid-associated resistance. We isolated a large group of *Enterobacteriaceae* resistant to IC concentrations from both raw sewage (*n* = 89) and activated sludge (*n* = 112, in order to assess the potential influence of the activated sludge process on (a) the relative abundance of IC resistance; (b) the scope of resistance to other antibiotics; and (c) the abundance of selected mobile genetic elements.

Interestingly, although no significant increase in the level of IC-resistant isolates was observed when comparing the sludge to the raw sewage, the sludge IC isolates were characterized by a significantly higher ratio of *qnr*-positive variants, indicating that these plasmid-associated elements enhance the fitness of the *Enterobacteriaceae* isolates in the activated sludge. This may be due to the *qnr* genes themselves, or alternatively to additional genes that are carried on mobile genetic elements that also harbor the *qnr* genes. The acquisition of plasmids carrying *qnr* genes increases the MIC of ciprofloxacin for wild-type *E. coli* J53 from 0.016 to 0.25 μg/ml (Robicsek et al., 2006) and therefore, it may be assumed that *qnr* confers a selective advantage to bacteria residing in the sludge, where ciprofloxacin concentrations of up to 0.05 μg/mg have been detected (Golet et al., 2002; McClellan and Halden, 2010).

We discovered that although the activated sludge process did not appear to select for a specific resistance, there appeared to be an increase in the degree of multi-resistance of the sludge IC isolates relative to the raw sewage isolates. However, with the exception of nalidixic acid, the increase of IC sludge vs. raw sewage isolates resistant to additional antibiotics was not statistically significant, and therefore a larger pool of isolates will need to be screened before this hypothesis can be established.

Typically, plasmid-mediated quinolone resistance was thought to be associated with class-1 integrons (Robicsek et al., 2006; Lapierre et al., 2008). However, the increased abundance of *qnr* elements observed in the sludge isolates relative to the raw sewage appears to stem from *qnr* elements that were not *intl-1* positive (**Figure 6**), suggesting that the *qnr* genes that proliferate in the sludge IC isolates are not associated with class-1 integrons. In total, raw sewage IC isolates that harbored the *intl-1* gene had a probability of 81.5% to carry a *qnr* gene, whereas only 75% of the *intl-1* positive sludge IC isolates carried a *qnr* gene (I^+^Q^+^). If the integron was indeed the genetic platform on which *qnr* resides, one would expect to see a correlated decrease between *qnr* and *intl-1* in class-1 integron negative isolates in both raw sewage and sludge. In reality, while only 29% of *intl-1* negative raw sewage isolates harbored a *qnr* gene, 75% of *intl-1* negative sludge isolates were. In contrast, 67% of the *qnr*-positive raw sewage isolates were also positive for *intl-1*, whereas only 50% of the sludge isolates did. Furthermore, only 16% of the *qnr*-negative raw sewage IC isolates carried the *intl-1* gene, whereas 50% of the *qnr*-negative sludge isolates did. This strongly suggests that *qnr* genes are not essentially associated with type-1 integrons, and that the dynamics of *qnr* in sludge may differ from that in raw sewage. A few studies have shown that the association between qnr and class-1 integrons is not mandatory. For example, Richter et al. (2010) identified an association between a *qnr* gene and a Tn2012 transposon. This novel transposon was comprised of a transposase gene (ISEcp1C) and a *qnr*B19 gene that were inserted into a Tn1721 transposon, a non-conjugative transposon from the Tn3 family. This genetic structure allows the *in vitro* dissemination of the *qnr*B19 gene into conjugative strains (Cattoir et al., 2008).

In conclusion, as depicted from our data, the presence of *qnr* (both with and without corresponding *intl-1* genes) is favored in the WWTP process irrespective of class-1 integron presence, thereby eliminating the suggested linkage between class-1 integrons and plasmid-associated quinolone resistance. This is of particular interest since it was previously described that qnr tend to be found in correlation to the class-1 integrons, and could suggest a different, negative correlation between those two genetic entities. Future research will need to screen the flanking regions of these *qnr*s in order to determine which mobile genetic elements the *qnr*s are located on, and if there are ubiquitous flanking genes that potentially bestow a selective advantage in the activated sludge environment.

## Conflict of Interest Statement

The authors declare that the research was conducted in the absence of any commercial or financial relationships that could be construed as a potential conflict of interest.
